# Association of Muscular Fitness and Body Fatness with Cardiometabolic Risk Factors: The FUPRECOL Study

**DOI:** 10.3390/nu10111742

**Published:** 2018-11-12

**Authors:** María Correa-Rodríguez, Robinson Ramírez-Vélez, Jorge Enrique Correa-Bautista, Rocío del Pilar Castellanos-Vega, Florencio Arias-Coronel, Katherine González-Ruíz, Hugo Alejandro Carrillo, Jacqueline Schmidt-RioValle, Emilio González-Jiménez

**Affiliations:** 1 Departamento de Enfermería, Facultad de Ciencias de la Salud, Avda. De la Ilustración, 60, Universidad de Granada, 18016 Granada, Spain; macoro@ugr.es (M.C.-R.); jschmidt@ugr.es (J.S.-R.); emigoji@ugr.es (E.G.-J.); 2 Centro de Estudios para la Medición de la Actividad Física CEMA, Escuela de Medicina y Ciencias de la Salud, Universidad del Rosario, Bogotá 111221, Colombia; jorge.correa@urosario.edu.co; 3 CORPS Group, Faculty of Health Sciences, University of Boyacá, Boyacá 153610, Colombia; dpcastellanos@uniboyaca.edu.co (R.d.P.C.-V.); farias@uniboyaca.edu.co (F.A.-C.); 4 Grupo de Ejercicio Físico y Deportes, Vicerrectoría de Investigaciones, Universidad Manuela Beltrán, Bogotá DC 110231, Colombia; katherine.gonzalez@docentes.umb.edu.co; 5 Grupo GRINDER, programa de Educación Física y Deportes, Universidad del Valle, Santiago de Cali 76001, Colombia; hugo.carrillo@correounivalle.edu.co; 6 Grupo Interdisciplinario de Estudios en Salud y Sociedad (GIESS), Institución Universitaria Escuela Nacional del Deporte, Santiago de Cali 76001, Colombia

**Keywords:** muscular fitness, body fatness, fat mass index, cardio-metabolic risk, young adults

## Abstract

This study investigated the associations of muscular fitness and various indicators of body fatness with cardio-metabolic risk factors and determined the muscular strength and body fatness thresholds for detecting a high risk of cardio-metabolic dysfunction in young adults. A cross-sectional study was conducted on 1798 collegiate students (61.5% females, mean age 20.5 years). Muscular fitness was determined by using a handgrip strength test and normalized grip strength (NGS = handgrip (kg)/body mass (kg)). Body mass index (BMI), waist circumference (WC), percentage of fat mass (BF%), fat-mass index (FMI), and waist-to-height ratio (WHR) were also included as body fatness measurements. A high cardio-metabolic risk cluster was derived by assessing triglycerides, low-density lipoprotein (LDL) cholesterol, high-density lipoprotein (HDL) cholesterol, fasting glucose, and blood pressure. Logistic regression models showed that men and women with lower NGS had an increased cardio-metabolic risk odds ratio (OR) = 1.8, 95% confidence interval (CI) 1.1 to 2.9, *p* = 0.006, and OR = 1.6, 95% CI 1.0 to 2.5, *p* = 0.036, respectively). In both sexes, higher levels of all fatness parameters were also associated with increased cardio-metabolic risk (*p* < 0.001). In both men and women, high FMI had the highest OR for clustered risk (OR = 4.7, 95% CI 2.6 to 8.4, and OR = 7.3, 95% CI 3.4 to 9.7, *p* < 0.001, respectively). Combined analysis showed that unfitness (lower NGS) and high fat had the highest OR for WC and FMI in men and women, respectively (OR = 5.5, 95% CI 2.6 to 11.4, OR = 7.7, 95% CI 2.3 to 15.8, *p* < 0.01). Muscular strength and body fatness are independently and jointly associated with increased cardiometabolic risk in young adults, which suggests that both are predictor variables for this.

## 1. Introduction

Metabolic and cardiovascular disturbances such as visceral adiposity, dyslipidemia, elevated blood pressure, and impaired insulin metabolism have been identified as risk factors for cardiovascular disease (CVD) and all-cause and CVD mortality. The fastest rise in CVD mortality has been observed in low-income and middle-income countries [1]. In Colombia, national overweight and obesity trends have been adversely affected by Westernised dietary habits and chronic physical inactivity [2]. Consequently, adiposity excess is a key contributor to the clustering of unfavorable cardio-metabolic risk factor levels in youth populations [3]. 

Muscular strength, as determined with a handgrip dynamometer, is becoming increasingly recognized as a predictor of all-cause mortality in healthy populations [4]. In fact, consistent evidence supports the idea that muscular fitness is a predictor of cardio-metabolic diseases and mortality [5,6]. In recent years, research has shown that muscle strength is negatively associated with metabolic syndrome in young adults [7,8,9,10,11]. Additionally, in a prospective study (20-year follow up of young adults), Fraser et al. [12] showed that those in the lowest third tertile for strength were independently associated with adult metabolic syndrome. The physiological pathways relating muscular fitness with cardio-metabolic risk place muscle mass as the main driver of this relationship since skeletal muscle is the principal site of insulin-mediated glucose uptake, which is closely related to muscular fitness [13]. 

On the other hand, there is growing evidence supporting the hypothesis that both general and central adiposity have a causal effect on cardiovascular events [14,15,16,17,18]. Fatness is defined as excess fat in places classically associated with adipose tissue storage and may contribute to inflammation and insulin resistance [19]. Both situations are related to disorders of other organs and tissues, e.g., abdominal fat and skeletal muscles [20]. These organs and tissues play a central role in glucose and lipid metabolism [21]. Changed body composition arising from obesity-related reduced skeletal muscle mass and increased visceral fat, known as ‘sarcopenic obesity’, aggravates insulin resistance and, thus, seems to be closely related to the onset and progression of CVD mediated by the abnormal metabolism of glucose and lipids [22]. In longitudinal data, childhood waist circumference (WC) has been reported as one of the strongest predictors of metabolic syndrome in adulthood [23].

A better understanding of how muscular fitness and fatness relate to other cardio-metabolic risk factors has the potential to improve health promotion efforts. To date, there is limited evidence on the influence of muscle strength and single and combined fatness measurements on clustered cardio-metabolic risk in young adults. 

Accordingly, the aim of this study was two-fold: (i) to investigate the associations of muscular fitness and various body fatness indicators with cardio-metabolic risk factors and (ii) to determine the cut-off muscular strength and body fatness levels for detecting a high risk of cardio-metabolic dysfunction in Colombian university students.

## 2. Methods

### 2.1. Study Design and Sample Population

We used secondary data from the cross-sectional Fuprecol Study adolescents and young adults, which investigated the association between muscular strength and metabolic risk factors in Colombian collegiate students. We recently published a complete description of the Fuprecol study’s design and methods and the primary outcomes for our current cohort [24]. The students were enrolled in public or private universities from three distinct areas of Colombia: the capital district of Bogota (Cundinamarca), Tunja (Boyacá), and Santiago de Cali (Valle del Cauca). Of this group, 1798 collegiate students (61.5% females) had valid data for body composition and all the components of the cardio-metabolic variables. 

The Committee for Human Research Ethics of Universidad Manuela Beltrán (UMB Code Nº 01-1802-2013) approved the study, which complied with Colombian laws (Declaration and Resolution 8430 of the Ministry of Health of Colombia, 1993) and the principles of the Declaration of Helsinki. All participants and their parents/guardians provided their written, informed consent.

### 2.2. Muscular Strength 

We used a handgrip strength test (T.K.K. 5001, Grip-A, Takei, Japan) adjusted for each participant by sex and hand size as muscular fitness “*proxy*”. The participants were instructed to stand with their arms completely extended, squeezing the handgrip gradually and continuously as hard as they could for at least 2 s. The test was performed twice, alternating hands between tests. A 90-s rest period was provided between trials. The best score for each hand was recorded in kilograms. The handgrip score (kg) was calculated as the average of the left and right hands and then expressed per kilogram of body weight. The reproducibility of our data was *R* = 0.96. Intra-rater reliability was assessed by determining the intra-class correlation coefficient (0.98, CI 95% 0.97–0.99, *n* = 20, median age = 22.8 ± 1.4 years, 66.2 ± 5.4 kg, 1.67 ± 0.1 m, 24.9 ± 3.1 kg/m^2^). Since there is substantial covariance between strength capacity and body mass and the link between strength and the proportion of strength relative to body mass directly mediates both physical function and chronic health, handgrip strength was normalized as grip strength (NGS) per body mass, i.e., handgrip strength in kg/body mass in kg. 

### 2.3. Body Fatness Examination

Fatness and clinical examinations were carried out between 25 January 2015 and 30 March 2017 by trained personnel and followed standardized routines described in detail elsewhere [24]. Briefly, fat mass and the fat mass index were measured by using tetrapolar whole body impedance (Tanita Model BC-420^®^, TANITA Corporation, Tokyo, Japan). A detailed description of the bioelectrical impedance analysis (BIA) technique can be found in a previous study [24]. The corresponding intra-observer technical error (% reliability) of the measurements was 95%. WC (cm) was measured at the uppermost border of the iliac crest around the abdomen. When this point was not evident, WC was measured at the midpoint between the last rib and the iliac crest using a metal tape measure (Lufkin W606PM^®^, Allers Parsippany, NJ, USA) in accordance with the International Society for the Advancement of Kinanthropometry guidelines.

Body mass index (BMI) was calculated as (weight/height^2^) and was classified using the World Health Organization criteria (normal: 18.5 to 24.9 kg/m^2^, overweight: 25.0 to 29.9 kg/m^2^, and obese: ≥30 kg/m^2^). The waist-to-height ratio (WHR) was expressed as the ratio of WC (in cm) to height (in cm). For all measurements, the tape was positioned at a level parallel to the floor. The evaluation process was carried out by a team of professionals (four physical therapy professors) with extensive experience in anthropometric measurement. In addition, 2% of the sample was measured twice to ensure the quality of the measurements. The technical error of measurement (TEM) values were less than 2% for all anthropometric variables. 

### 2.4. Cardio-Metabolic Risk Factors

For blood measurements, the participants were asked to arrive in a fasting state by abstaining from exercise training, caffeine, nicotine, and alcohol 12 h before the clinical examination and continue their regular medication routines. Capillary blood samples (40 µL) were collected for determining serum biochemical parameters including triglycerides, low-density lipoprotein cholesterol (LDL-c), high-density lipoprotein cholesterol (HDL-c), and fasting glucose by using portable Cardiocheck^®^ equipment (Mexglobal SA, Parsippany, NJ, USA). 

The blood pressure was measured with an automatic monitor (Omrom^®^ HEM 705 CP, Health-care Co, Kyoto, Japan) following the recommendations of the European Heart Society (on the right arm, with the participants in a supine position, and after 10 min of rest). During the measurement, the participants were seated with the arm supported at the level of the heart.

The presence of cardio-metabolic risk factors was defined on the basis of diagnostic criteria for the metabolic disorders [25]: elevated triglyceride (TG) level (≥150 mg/dL) or pharmacological treatment for dyslipidemia, reduced HDL cholesterol level (<40 mg/dL in women and <50 mg/dL in men), or pharmacological treatment for dyslipidemia, elevated blood pressure (systolic ≥ 130 mm Hg and/or diastolic ≥ 85 mm Hg) or pharmacological treatment for hypertension, and an elevated fasting glucose level (≥100 mg/dL) or pharmacological treatment for diabetes. In addition, an elevated LDL cholesterol level (≥110 mg/dL) or pharmacological treatment for dyslipidemia was considered in the definition of high cardiometabolic risk, as suggested by the 2013 American College of Cardiology/American Heart Association guideline [25]. High cardio-metabolic risk was defined as the presence of two or more of these risk factors. Because WC is strongly correlated with other indices of fatness, WC was not considered in the definition of high cardio-metabolic risk to avoid biasing the results against the higher fatness groups, which was suggested by other studies [26,27]. 

### 2.5. Covariables

Data on smoking were collected via self-reported questionnaires (number of cigarettes smoked per day). The smoking status was determined as a non-smoker or quit smoking ≥12 months ago via personal interviews with the participants [28]. The physically active category was defined as ≥150 min of moderate activity per week at least three times per week [28]. Food consumption was assessed by the Kidmed index (Mediterranean Diet Quality Index) was used. The index is based on a 16-questions self-administered, which sustain the principles of the Mediterranean dietary patterns, as well as, those that undermine it. In this study, we divided participants into two groups: less or equal to 8 points (ideal healthy diet), and less or equal to 7 points (non-ideal healthy diet). 

### 2.6. Data Management

Participants were divided into the following groups according to the fatness parameters (BMI, WC, percentage of fat mass (%BF), fat-mass index (FMI), and WHR) and muscular fitness (fit and unfit): (i) Unfit and low fat; (ii) unfit and high fat; and (iii) fit and high fat as previously described [13]. 

### 2.7. Statistical Analysis

Descriptive statistics were computed and summarized. Continuous variables are reported using means and standard deviations (SD) and categorical variables are reported using proportions (%). The *t*-test was used to compare unadjusted means and the chi-square test (χ^2^) was used to explore sex-group differences. Receiver operating characteristic (ROC) curve analysis was used to estimate the optimal thresholds of NGS, BMI, WC, %BF, FMI, and WHR that best identify individuals with high cardio-metabolic risk (i.e., ≥2 cardio-metabolic risk factors present) [29]. All combinations of sensitivity and 1 − specificity that can be realized by incrementally changing the cut-off values of NGS, BMI, WC, %BF, FMI, and WHR were summarized as the areas under the ROC curves (AUC) with 95% CIs for each parameter. Optimal thresholds for each variable were estimated by the Youden Index [30] as the value producing the combination of sensitivity and specificity closest to 1 (i.e., perfect test). Sensitivity, specificity, positive likelihood ratio (LR+), negative likelihood ratio (LR-), positive predictive value (PPV), and negative predictive value (NPV) of the CRF were calculated at all possible cut-points to find the optimal value. 

Logistic regression analyses were used to estimate adjusted odds ratios (ORs) and 95% CIs for having high cardio-metabolic risk, according to independent and combined categories of NGS, BMI, WC, %BF, FMI, and WHR. All analyses were performed separately for men and women. Statistical programs MedCalc 16.8.4^®^ (MedCalc software bvba, Ostend, Belgium) and SPSS (IBM software, v.22.0, SPSS, Inc., Chicago, IL, USA) were used for all analyses and a value of *p* < 0.05 was considered statistically significant.

## 3. Results

### 3.1. Study Participants

The descriptive characteristics of the participants are shown in Table 1. The mean age of the sample was 20.5 (3.1) years and there were more women (61.5%) than men (38.5%). The men had higher body weights, heights, and WCs than the women (*p* < 0.001) while the women had higher BF% and FMI (*p* < 0.001). Furthermore, systolic and diastolic blood pressures were higher in men than in women (*p* < 0.001). For cardio-metabolic parameters, the men had higher TG levels while the women had higher HDL cholesterol and glucose levels (*p* < 0.05). The women had a similar metabolic risk (*p* = 0.826) and lower physical activity levels than the men (*p* < 0.001). Lastly, handgrip and NGS were significantly higher in men than in women (*p* < 0.001).

### 3.2. Association between Fitness and Fatness Parameters with Cardiometabolic Risk Factors

A logistic regression analysis of fitness, fatness parameters, and highly clustered risk after adjusting by age, alcohol consumption, tobacco use, and dietary intake is shown in Table 2. Men and women with lower NGS had increased cardio-metabolic risk (OR = 1.8, 95% CI 1.1 to 2.9, *p* = 0.006, and OR = 1.6, 95% CI 1.0 to 2.5, *p* = 0.036, respectively). In both sexes, higher levels of all fatness parameters (BMI, WC, %BF, FMI, and WHR) were also associated with increased cardio-metabolic risk (*p* < 0.001). Note that, in both men and women, high FMI had the highest OR for clustered risk (OR = 4.7, 95% CI 2.6 to 8.4, *p* < 0.001, and OR = 7.3, 95% CI 3.4 to 9.7, *p* < 0.001, respectively). 

To further explore the association of muscular strength and fatness measurements with high cardio-metabolic risk, the combined effects were examined (Table 3). For all fatness parameters, the unhealthiest profiles (unfitness and high fat) in both genders were associated with increased cardio-metabolic risk adjusted for confounding factors (*p* < 0.001). Unfitness and high fat profiles had the highest OR for WC in men and for FMI in women (OR = 5.5, 95% CI 2.6 to 11.4, *p* ≤ 0.001, and OR = 7.7, 95% CI 2.3 to 15.8, *p* = 0.001, respectively).

### 3.3. Optimal Cut-Off Metabolic Syndrome Screening Value

The ROC curve analyses diagnosing how well fitness and fatness parameters identify high cardio-metabolic risk in men and women are presented in Table 4. The ROC analyses showed that fitness and fatness parameters could be used to identify highly clustered cardio-metabolic risk in young adults. 

For men, the NGS cut-off value of 0.56 provided a sensitivity of 56% and a specificity of 65%. In fatness parameters, the BMI cut-off value of 24.7 kg/m^2^ provided a sensitivity of 43% and a specificity of 81% and the WC cut-off value of 83.0 cm provided a sensitivity of 39% and a specificity of 85%. The ROC curve for BF% was also obtained by using a cut-off value of 19.2%, a sensitivity of 42%, and a specificity of 83%. Furthermore, the FMI cut-off value of 4.78 kg/m^2^ provided a sensitivity of 42% and a specificity of 83% and the WHR cut-off value of 0.49 provided a sensitivity of 32% and a specificity of 88%.

For women, the NGS cut-off value of 0.39 provided a sensitivity of 49% and a specificity of 65%. For fatness parameters, the BMI cut-off value of 26.6 kg/m^2^ provided a sensitivity of 28% and a specificity of 89%. The WC cut-off value of 79.4 cm provided a sensitivity of 46% and a specificity of 77%. The ROC curve for the BF% was also obtained by using a cut-off value of 32.9%, a sensitivity of 28%, and a specificity of 88%. Furthermore, the FMI cut-off value of 7.92 kg/m^2^ provided a sensitivity of 40% and a specificity of 83% and the WHR cut-off value of 0.50 provided a sensitivity of 22% and a specificity of 91%.

## 4. Discussion

This study investigated the relationships between muscle strength, fatness parameters, and high cardio-metabolic risk in a large population of young Colombian adults. The findings indicate that muscle strength and fatness parameters are both independently and jointly associated with clustered cardio-metabolic risk, which supports the hypothesis that muscular unfitness and higher fatness profiles are predictors of cardio-metabolic risk in early adulthood.

Muscle strength is increasingly recognized as an emerging risk factor for major causes of death in young adulthood including cardiovascular disease [4,31,32]. Our results show that lower muscle strength levels were independently associated with a cardio-metabolic risk profile in young adults. This finding supports a protective effect of muscular strength on clustered cardio-metabolic risk. Consistent with these findings, muscle fitness has been inversely associated with the lipid-metabolic profile and metabolic syndrome risk in young people [7,8,9,10,11]. Similarly, Jurca et al. reported that, in a large population of adult men, muscular strength was associated with metabolic syndrome prevalence independently of aerobic fitness [33]. A recent longitudinal study with a 20-year follow up in young adulthood showed that childhood muscular fitness can be used to predict adult metabolic syndrome [12]. 

Therefore, this study, which is in line with the results mentioned above, supports the inclusion of strength training in physical activity recommendations since it may provide unique cardio-metabolic health benefits. It is possible that resistance training may confer protection against cardiovascular risk beyond that seen for cardiorespiratory fitness. The exact mechanisms through which high muscle fitness independently reduces cardio-metabolic risk have not been fully elucidated, but the increased muscular fitness might produce metabolic and structural changes that improve muscle insulin sensitivity and glycaemic control [34,35]. It has been hypothesized that the apparent protective effect of muscular strength against high-risk clustered cardio-metabolic risk may not be a function of maximal muscular strength but may reflect better metabolic homeostasis resulting from regular participation in physical resistance activities [36]. 

In contrast, adiposity has been shown to be related to increased cardio-metabolic risk [17,23,37]. We observed that all body fatness measurements included in this study were moderately associated with the clustered cardio-metabolic risk profile. These results are consistent with previous studies that have reported an association between high body fatness and metabolic syndrome risk [23]. To date, this study has examined the value of several body fatness measurements including BMI, WC, BF%, FMI, and WHR for predicting cardio-metabolic risk in young adults. Interestingly, it should be noted that FMI was found to be the best predictor of high cardio-metabolic risk in women. These results indicate that, in addition to being an effective obesity screening tool [24], FMI are a useful indicator for predicting cardio-metabolic risk in early adulthood. In line with previous studies, WC and BF% appear to be the most important factor for predicting clustered metabolic risk in men [23]. In this sense, it was observed that the positive predictive value in body fatness measurements was important for both sexes (range from 48.7% to 61.9% in men and range from 44.7% to 61.0% in women, respectively), which suggests a greater number of false positives. This positive predictive value is directly related to the low prevalence of the highly clustered cardio-metabolic risk in the overall sample (~36%). However, this cut-off point can only be applied to populations where the condition has a similar prevalence to the population tested or to individuals with a similar risk of a positive result. This suggestion should always be applied with caution in clinical practice due to population differences having a huge impact on their interpretation.

To date, few studies have investigated the combined effect of muscular fitness and fatness since most previous reports have focused on the combined association of muscle strength and aerobic capacity with metabolic syndrome risk [7,8,9,10,33,38]. Our results are consistent with some recent findings indicating that the unfit and high body fat group had the highest cardio-metabolic risk when compared to the unfit and low body fat as well as the fit and low body fat groups. In addition, we found that the unfit and low-fat group is less risky than the fit and high-fat group. In this context, a previous study demonstrated that both increased body fatness and decreased muscular strength were associated with metabolic risk in young college students, which highlights the fact that being physically fit can confer an added benefit on a healthy body composition [39]. Similarly, Jekal et al. [40] found that adolescents who were in the high-fat-high-fit group had a significantly lower CVD risk score than those who were high-fat-low-fit.

Eisenmann et al. [41] also reported that both fatness and fitness may be considered when assessing CVD risk factors in young people especially among high-fat youth. The combined observed associations of muscular fitness and fatness with cardio-metabolic health suggest that both indicators may be related to common benefits. Resistance training may increase muscle quantity and insulin action and reduce adipose tissue. Since both the predictors found in this study are modifiable, our findings support the relevance of promoting strength training in addition to preventing body fat accumulation to improve the cardio-metabolic health profile in young people [42]. 

The cut-points found in the present study were used to classify the sample by indicators of grip strength and fatness parameters in identifying high cardio-metabolic risk. The thresholds established to detect high risk in young people were the following: NGS ≥ 0.56 kg (men) and ≥0.39 kg (women), BMI ≥ 24.7 kg/m^2^ (men), and ≥26.6 kg/m^2^ (women), WC ≥ 83.0 cm (men) and ≥79.4 cm (women), BF% ≥ 19.2% (men) and ≥32.9% (women), FMI ≥ 4.78 kg/m^2^ (men) and ≥7.92 kg/m^2^ (women), and WHR ≥ 0.49 (men) and ≥0.50 (women). 

Some limitations of this study should also be addressed. First, the sample is non-probabilistic. Second, the cross-sectional design does not allow us to explain causality. Thus, future prospective analysis is necessary to determine causal links between the combination of muscular fitness, body fatness, and cardio-metabolic risk. Third, muscle fitness was assessed only by measuring isometric strength and explosive and endurance strength were not assessed. However, isometric handgrip dynamometry is considered a reliable method for assessing strength [43]. Moreover, evidence from the literature suggests that previous fatness cut-points were also developed by using cross-sectional samples [24]. The indicators analyzed (BMI, WHR, and WC) are considered less accurate for estimating body fat than densitometric or imaging techniques to identify fatness. However, such indicators are recommended when it comes to large samples due to low operating cost and easy application. Moreover, the combination of the five indicators used in the present study can be considered an adequate strategy because it classified young adults as general and fatness simultaneously. Fifth, although it should be of interest to analyze the direction of the association in a fit and low-fat group, we have not included it in our study. Lastly, since our study population comprised a well-characterized cohort of young Colombian adults, the results presented in this scenario may not be generalizable to other ethnicities or age ranges. 

Additional strengths of this study include the measurements of both strength and body fatness using highly reliable measuring devices. Furthermore, it should be noted that, in this study, several standardized fatness indicators including BMI, WC, BF%, FMI, and WHR were objectively measured. Additionally, since we collected lifestyle factors, the muscular fitness and body fatness analysis were extensively adjusted for relevant confounding variables including food consumption. Lastly, highly standardized procedures have been developed within the Fuprecol study to avoid the measurement bias.

## 5. Conclusions

In summary, we found that muscular strength and body fatness measurements are both independently and jointly associated with increased cardio-metabolic risk in young adults. This suggests that both are predictor variables related to cardio-metabolic dysfunction in young people. These findings have clinical significance since we highlight the importance of including muscular fitness and fatness testing in health-monitoring systems in early adulthood for the primary prevention of metabolic disorders.

## Figures and Tables

**Table 1:** Characteristics among a sample of college students from Colombia.

Characteristics	Men (*n* = 692)	Women (*n* = 1106)	*p* Value
Age (years)	20.5 (3.2)	20.5 (2.9)	0.077
**Anthropometric and fatness parameters**			
Weight (kg)	68.9 (12.2)	58.7 (10.3)	<0.001
Height (cm)	172.3 (6.6)	159.0 (5.8)	<0.001
WC (cm)	78.2 (9.3)	71.5 (8.0)	<0.001
High fat N, (%) *	77 (11.1)	153 (13.8)	0.071
BF%	15.6 (6.5)	27.0 (7.2)	0.002
High fat N, (%) *	58 (8.3)	59 (5.3)	0.018
FMI (kg/m^2^)	3.8 (2.2)	6.5 (2.7)	<0.001
High fat N, (%) *	62 (8.9)	56 (5.1)	0.002
BMI status	23.1 (3.6)	23.2 (3.7)	0.089
High fat N, (%) *	178 (25.6)	296 (26.7)	0.616
WHR (cm)	0.463 (0.05)	0.451 (0.05)	<0.001
High fat N, (%) *	123 (17.4)	173 (15.3)	0.216
**Blood pressure**			
Systolic blood pressure (mmHg)	120.2 (12.9)	111.2 (11.1)	<0.001
Diastolic blood pressure (mmHg)	74.1 (11.4)	71.7 (9.3)	<0.001
**Cardio-metabolic parameters**			
Triglycerides (mg/dL)	93.7 (48.5)	88.5 (45.3)	0.017
LDL cholesterol (mg/dL)	81.0 (26.0)	87.9 (26.1)	0.386
HDL cholesterol (mg/dL)	39.5 (10.6)	43.9 (12.8)	<0.001
Glucose (mg/dL)	84.8 (11.9)	86.0 (11.5)	0.010
**High metabolic risk N (%) ^#^**	253 (36.5)	398 (36.0)	0.826
**Life-style**			
Tobacco (≥10 cigarettes per week) N, (%) *	199 (28.7)	210 (19.0)	0.548
Alcohol (≥1 times per week) N, (%) *	378 (54.6)	430 (38.9)	0.451
Physical activity (>150 min per week) N, (%) *	243 (35.1)	222 (20.1)	0.001
**Adherence to a MedDiet (food consumption)**			
Optimal adherence N, (%) *	76 (11.0)	124 (11.1)	0.241
**Muscular strength**			
Handgrip (kg)	39.4 (7.1)	24.0 (4.9)	<0.001
NGS	0.582 (0.11)	0.416 (0.09)	<0.001

Continuous variables are reported by using means and standard deviations (SD) and categorical variables are reported using proportions N, (%) *. The *t*-test was used to compare unadjusted means and the chi-square test (χ^2^) * was used to explore sex-group differences. NGS, normalized grip strength (Handgrip (kg)/body mass (kg)), BMI, body mass index, WC, waist circumference, BF%, Body fat percentage, FMI, fat mass index, WHR, Waist to height ratio, LDL, low-density lipoprotein cholesterol, HDL, high-density lipoprotein cholesterol, MedDiet, KIDMED Mediterranean Diet Quality Index, ^#^ High cardio-metabolic risk defined as the presence of at least two abnormalities.

**Table 2:** Associations between fitness and fatness parameters with high cardio-metabolic risk.

Parameter	Men (*n* = 692)			Women (*n* = 1106)		
	OR	95% CI	*p* Value	OR	95% CI	*p* Value
Low NGS	1.8	1.1–2.9	0.006	1.6	1.0–2.5	0.036
High body mass index	3.4	2.3–4.9	<0.001	3.5	2.4–5.2	<0.001
High waist circumference	4.5	2.7–7.6	<0.001	4.1	2.5–6.5	<0.001
High body fat percentage	4.5	2.4–8.1	<0.001	6.4	3.1–8.1	<0.001
High fat mass index	4.7	2.6–8.4	<0.001	7.3	3.4–9.7	<0.001
High WHR	3.8	2.5–5.9	<0.001	4.0	2.5–6.4	<0.001

NGS, normalized grip strength (Handgrip (kg)/body mass (kg)). WHR, Waist to height ratio. Analysis adjusted by age, alcohol, tobacco, and food consumption.

**Table 3:** Combined effects of muscular fitness and fatness parameters on high cardio-metabolic risk.

Parameter	Men (*n* = 692)			Women (*n* = 1106)		
	OR	95% CI	*p* Value	OR	95% CI	*p* Value
**Body mass index**						
Unfit and low fat	1.0	0.5–2.1	0.858	0.7	0.2–1.7	0.441
Unfit and high fat	4.6	2.5–8.5	<0.001	3.8	2.1–6.6	<0.001
Fit and high fat	2.9	1.9–4.5	<0.001	3.3	2.1–5.1	<0.001
Fit and low fat	1.0 (Reference)			1.0 (Reference)		
**Waist circumference**						
Unfit and low fat	1.0	0.6–1.9	0.763	1.1	0.6–2.0	0.727
Unfit and high fat	5.5	2.6–11.4	<0.001	4.1	2.0–8.1	<0.001
Fit and high fat	3.6	1.7–7.4	0.001	4.1	2.2–7.5	<0.001
Fit and low fat	1.0 (Reference)			1.0 (Reference)		
**Body fat percentage**						
Unfit and low fat	1.4	0.8–2.4	0.192	1.1	0.6–1.93	0.655
Unfit and high fat	4.7	2.1–10.7	<0.001	7.4	2.9–18.9	<0.001
Fit and high fat	4.6	1.9–10.9	<0.001	5.3	1.7–16.9	0.004
Fit and low fat	1.0 (Reference)			1.0 (Reference)		
**Fat mass index**						
Unfit and low fat	1.2	0.7–2.1	0.435	1.133	0.6–1.94	0.650
Unfit and high fat	4.8	2.3–10.2	<0.001	7.751	2.3–15.8	0.001
Fit and high fat	4.8	1.9–11.9	0.001	7.332	2.8–18.9	<0.001
Fit and low fat	1.0 (Reference)			1.0 (Reference)		
**WHR**						
Unfit and low fat	1.1	0.7–1.6	0.661	1.1	0.6–2.3	0.757
Unfit and high fat	4.1	2.1–10.1	<0.001	4.1	2.1–9.1	<0.001
Fit and high fat	2.6	1.6–5.31	<0.001	3.1	2.9–5.1	<0.001
Fit and low fat	1.0 (Reference)			1.0 (Reference)		

Analysis adjusted by age, alcohol, tobacco, and food consumption. WHR, Waist to height ratio.

**Table 4:** Parameters of the ROC curve analysis for the diagnostic performance of fitness and fatness parameters in identifying high cardio-metabolic risk in men and women.

	Parameters	NGS	BMI	WC	BF%	FMI	WHR
**Men** **(*n* = 692)**	AUC (SE)	0.617 (0.022)	0.662 (0.021)	0.669 (0.021)	0.681 (0.021)	0.679 (0.021)	0.650 (0.022)
	95% CI	0.580 to 0.653	0.626 to 0.697	0.632 to 0.703	0.645 to 0.715	0.643 to 0.714	0.613 to 0.685
	*p*-value	<0.0001	<0.0001	<0.0001	<0.0001	<0.0001	<0.0001
	Cut-off	0.56	24.72	83.0	19.2	4.78	0.49
	Sensitivity (95% CI)	56.86 (50.5 to 63.0)	43.36 (37.2 to 49.7)	39.37 (33.3 to 45.7)	42.58 (36.4 to 48.9)	42.97 (36.8 to 49.3)	32.68 (26.9 to 38.8)
	Specificity (95% CI)	65.70 (61.1 to 70.1)	81.92 (78.0 to 85.4)	85.39 (81.8 to 88.5)	83.60 (79.8 to 86.9)	83.15 (79.3 to 86.5)	88.54 (85.2 to 91.3)
	+LR (95% CI)	1.66 (1.4 to 2.0)	2.40 (1.9 to 3.1)	2.70 (2.1 to 3.5)	2.60 (2.0 to 3.3)	2.55 (2.0 to 3.3)	2.85 (2.1 to 3.9)
	−LR (95% CI)	0.66 (0.6 to 0.8)	0.69 (0.6 to 0.8)	0.71 (0.6 to 0.8)	0.69 (0.6 to 0.8)	0.69 (0.6 to 0.8)	0.76 (0.7 to 0.8)
	%PPV (95% CI)	48.7 (42.9 to 54.5)	57.8 (50.5 to 64.9)	60.6 (52.7 to 68.1)	59.9 (52.4 to 67.1)	59.5 (52.0 to 66.6)	61.9 (53.2 to 70.2)
	%NPV (95% CI)	72.7 (68.1 to 77.0)	71.7 (67.6 to 75.5)	71.2 (67.1 to 75.0)	71.7 (67.6 to 75.5)	71.7 (67.6 to 75.6)	69.7 (65.8 to 73.5)
**Women** **(*n* = 1106)**	AUC (SE)	0.664 (0.017)	0.662 (0.016)	0.653 (0.017)	0.662 (0.017)	0.664 (0.017)	0.652 (0.017)
	95% CI	0.564 to 0.683	0.633 to 0.689	0.625 to 0.681	0.633 to 0.690	0.636 to 0.692	0.624 to 0.680
	*p*-value	<0.0001	<0.0001	<0.0001	<0.0001	<0.0001	<0.0001
	Cut-off	0.39	26.61	79.40	32.90	7.92	0.50
	Sensitivity (95% CI)	49.26 (44.3 to 54.2)	28.08 (23.8 to 32.7)	46.42 (41.5 to 51.4)	28.89 (24.5 to 33.6)	40.59 (35.8 to 45.6)	22.72 (18.7 to 27.1)
	Specificity (95% CI)	65.50 (61.9 to 69.0)	89.86 (87.4 to 92.0)	77.33 (74.1 to 80.3)	88.41 (85.8 to 90.7)	83.08 (80.1 to 85.8)	91.38 (89.1 to 93.3)
	+LR (95% CI)	1.43 (1.2 to 1.6)	2.77 (2.1 to 3.6)	2.05 (1.7 to 2.4)	2.49 (1.9 to 3.2)	2.40 (2.0 to 2.9)	2.63 (2.0 to 3.5)
	−LR (95% CI)	0.77 (0.7 to 0.9)	0.80 (0.7 to 0.9)	0.69 (0.6 to 0.8)	0.80 (0.8 to 0.9)	0.72 (0.7 to 0.8)	0.85 (0.8 to 0.9)
	%PPV (95% CI)	44.7 (40.1 to 49.5)	61.0 (53.6 to 68.0)	53.6 (48.2 to 58.9)	58.5 (51.3 to 65.4)	57.5 (51.6 to 63.4)	59.7 (51.5 to 67.6)
	%NPV (95% CI)	69.5 (65.9 to 72.9)	68.9 (65.8 to 71.9)	71.9 (68.6 to 75.1)	68.7 (65.6 to 71.7)	71.2 (68.0 to 74.3)	67.7 (64.7 to 70.7)

NGS, normalized grip strength (Handgrip (kg)/body mass (kg)), BMI, body mass index, WC, waist circumference, BF%, Body fat percentage, FMI, fat mass index, and WHR, Waist to height ratio. AUC: area under the curve, SE: standard error, CI: confidence interval, LR (+): positive likelihood ratio, LR (−): negative likelihood ratio, PPV: positive predictive value, and NPV: negative predictive value.
